# Planning for classroom physical distancing to minimize the threat of COVID-19 disease spread

**DOI:** 10.1371/journal.pone.0243345

**Published:** 2020-12-04

**Authors:** Alan T. Murray

**Affiliations:** Department of Geography, University of California, Santa Barbara, CA, United States of America; University of Missouri Columbia, UNITED STATES

## Abstract

The coronavirus disease 2019 is a respiratory illness spread between people. A primary weapon for reducing or eliminating this disease involves physical distancing to thwart transmission. Efforts to keep the economy moving include enacting physical distancing strategies that will increase the safety of workplaces, schools, businesses, etc. Given that education is a critical economic sector that impacts essentially all other sectors in some way, this paper details a planning approach for classroom physical distancing supported by spatial optimization. Devising a configuration of desks and/or workspaces that are physically distant is a type of dispersion problem that can be formalized mathematically and solved. Planning efforts for a university campus serve to illustrate how spatial optimization can support safety enhancements.

## Introduction

It is broadly known that coronavirus disease 2019 (COVID-19) is a respiratory illness primarily spread between people. The COVID-19 pandemic has been devastating, straining our health care systems, disrupting the economy and impeding social interaction. While vaccines and therapeutics offer a likely return to normal life activities, much success in reducing transmission and spread of COVID-19 has been demonstrated in many countries and communities through the implementation of basic practices and behaviors, including frequent washing hands, avoiding close contact between people (particularly outside of the same household), wearing masks that cover the nose and mouth, and cleaning / disinfecting common areas and frequently touched surfaces. Most government public health agencies recognize this, such as the U.S. Centers for Disease Control and Prevention [1], California Department of Public Health [2] and Santa Barbara County Public Health Department [3], advocating these strategies in strenuous terms.

A challenge in many different settings is avoiding close contact between people. This is commonly referred to as social or physical distancing. The U.S. Centers for Disease Control and Prevention [4] characterizes social / physical distancing as maintaining “… a safe space between yourself and other people who are not from your household”. The default recommendation is to stay at least 6 ft. from others for prolonged periods, as spread of COVID-19 is believed to occur through respiratory droplets and/or aerosol particles expelled when a symptomatic or asymptomatic person breathes, talks, coughs, sneezes, yells or sings [5]. While the science behind the spread of COVID-19 is still evolving, the foundations of physical distancing along with the wearing of masks in communities with low rates of infection make a return to certain activities possible. In this regard, schooling and work are particularly important for a range of reasons, with appropriate planning and precaution measures essential. Like most schools, colleges, workplaces, etc., administration and staff have and continue to need strategic plans to accommodate a resumption of activities. Central to much of this has been the need for detailed seating arrangements that allow for sufficient space between people. As an example, Virginia Tech [6] outlines physical distancing requirements, including six feet separation between individuals at all times, with examples for desk spacing in classrooms in order to maintain the six foot standard. The University of California, Santa Barbara, like most companies, organizations and colleges, is undertaking planning efforts for a return to in-person learning. Accordingly, UC Santa Barbara [7] provides detailed physical distancing requirements and procedures, along with seating plans for all rooms. The classroom physical distancing effort is being carried out by the Instructional and Study Space Workgroup, a cross-disciplinary workgroup convened by the Executive Vice Chancellor. The process involves a detailed evaluation of each classroom to determine seating and/or desk configuration strategies that provide for physical distancing.

The intent of this paper is to highlight how planning for classroom physical distancing to minimize the threat of COVID-19 disease spread can be supported by spatial analytics, and in particular spatial optimization. Devising a configuration of seating that is physically distant is a type of dispersion problem that can be formalized mathematically and solved. This provides a capability to formally prove quality in obtained findings. Two issues characterize dispersion goals in the case of seating in a classroom. One is deriving the maximum capacity and associated spatial configuration of individuals in a classroom that conforms to distancing requirements. The second is identifying the spatial configuration of individuals that provides the most spacing possible between each other beyond the minimum separation standard. The next section reviews dispersion modeling approaches in the context of classroom physical distancing. This is followed by the presentation of methods for carrying out seating capacity assessment that address dispersion goals. An examination of seating capacity for a large classroom is then detailed. The paper ends with discussion and conclusions.

## Background

The classroom seating challenge brought about by potential disease spread associated with COVID-19 is actually a type of dispersion problem. A general review of dispersion problems can be found in Church and Murray [8], but variants of particular interest here are:

P-1. Identify maximum number of individuals that can be accommodated where a separation of at least a distance of *R* between them is maintained
P-2. Distribute a fixed number of individuals, *p*, as far away from each other as possible

The parameter *R* represents a minimum spacing distance standard in the P-1 context. In the case of P-2, the parameter *p* is the number of individuals to be dispersed in a classroom. The spacing standard in P-1 is central in dictating the capacity of a room in accordance with permissible seating guidelines, such as the 6 ft. minimum discussed previously. Thus, *R* would be equal to 6 ft. in this planning context. A variation of the separation requirement is that highlighted by Virginia Tech [6], where the maximum number of students permissible in any classroom is 50 irrespective of the size of the room. This is stipulated in P-2. Thus, *p* would be equal to 50 in this planning context. There are in fact a number of relationships between P-1 and P-2. Of particular note is that a maximally dispersed solution identified in the context of P-2 may actually be infeasible with respect to P-1 if the distance between individuals is not at least *R*. This and other distinctions will be discussed and illustrated later in the paper, but both planning contexts reflect a desire to disperse individuals in order to promote safety. Further, it is possible that one general approach can be utilized to address both cases, as demonstrated later in the paper.

The research on methods and approaches to support dispersion planning is considerable. Early modeling work focusing on timber harvest scheduling was that of Thompson et al. [9] (see also [10]), where the capacity and location of forest management operations in an area were sought. The intent was to identify the most beneficial sites for timber extraction without any selected sites being too close to each other. A similar notion is behind the assessment of habitat carrying capacity for a region. Nesting sites for sandhill cranes were considered in Downs et al. [11] and spotted owls were of interest in Church et al. [12]. The fundamental question was how many nesting sites could be supported in an area and where they should be located given that these fauna require exclusive territory, with each nest separated from other nests. Considered in Grubesic and Murray [13] was the question of sex offender residency capacity for a region, where local ordinances prohibit those convicted of such an offense from residing near each other. Alcohol outlet saturation in a city was evaluated in Grubesic et al. [14] as laws requires businesses selling alcohol to be separated from each other. The unifying element of each of these different contexts is that a capacity of activities is sought, indicating how many and where individuals/businesses should be located in order to maintain separation requirements. This is precisely the intent behind COVID-19 physical distancing precautions in a classroom setting.

Beyond the different substantive and application contexts for seeking out dispersed configurations of individuals and/or operations, there has been much work on the formalization and solution of the resulting spatial optimization problem. Early work includes that of Padberg [15] and Nemhauser and Trotter [16], with explicit spatial attention in Moon and Chaudhry [17], Kuby [18], Erkut [19] and Murray and Church [20], among others. Various extensions of basic dispersion approaches have also been pursued, with Wei and Murray [21], Niblett and Church [22] and Murray et al. [10] reflecting more recent efforts.

## Methods

As suggested previously, the dispersion of individuals in a classroom setting can be conceived of in at least two ways, P-1 and P-2, depending on context and associated planning goals. Beginning with P-1, it is important to recognize that any seating arrangement that satisfies the *R* distance separation between individuals is feasible in terms of the intended safety standards seeking to avoid close contact between people. However, finding the largest number of individuals (and where they are to be seated) is a more challenging task. Indeed, this is a spatial optimization problem that has been of great interest and broadly applied in a range of substantive contexts over the past five decades.

Since P-1 is a spatial optimization problem, solution of a planning application can be approached in many ways. Perhaps not surprisingly, the COVID-19 pandemic highlights that human-centered techniques are regularly applied since seating arrangements are a common byproduct of most planning efforts. In particular, such approaches are characterized by a trial and error process, relying on human intuition and perception to devise a good seating arrangement. It is not uncommon for such an approach to be supported by visual maps and layouts in an interactive fashion. Indeed, packages like AutoCAD and Visio support this in some ways as do geographic information systems (GIS) such as ArcGIS, QGIS, etc. As long as room scaling and dimensions can be established, such approaches can be effective, especially when carried out by an analyst with experience, intuition and insights regarding effective and efficient seating patterns.

There is, however, a formalized approach that can be taken, involving the use of a mathematical model that is supported by computing technology. Consider the following notation:

*j* = index of potential seat locations

(*φ*_*j*_, *λ*_*j*_) = location coordinates of seat *j*

$d_{jj^{\prime }}=\sqrt{{\left(\varphi_{j}-\varphi_{j^{\prime }}\right)}^{2}+{\left(\lambda_{j}-\lambda_{j^{\prime }}\right)}^{2}}$

*R* = restriction distance between seats

Ω_*j*_ = {*j*′|*d*_*jj*′_≤*R*}

$X_{j}=\left\{ \begin{array}{l} 1\;if\;an\;individual\;is\;assigned\;to\;seat\;j\\ 0\;otherwise \end{array}\right.$

This notation serves as the foundation for specifying a model that reflects the intent of P-1. First, there are potential seat locations that are known in advance, referenced using the index *j*. Second, each potential seat location *j* has a specific geographic reference, (*φ*_*j*_, *λ*_*j*_). Third, with geographic reference to potential seats, it is possible to derive the distance between any pair of seats, *j* and *j*′, denoted *d*_*jj*′_. In this particular case the Euclidean distance metric is indicated. Fourth, with inter-seat distances *d*_*jj*′_, it is possible to identify those seats that are within the separation standard *R* of a given seat *j*, and this is reflected in the set Ω_*j*_. Finally, the remaining issue is a decision for each seat, whether to assign an individual to sit/work there or not. This is accomplished here using the decision variable *X*_*j*_ for each seat *j*.

Given this notation, a mathematical model can be structured to achieve the P-1 goal of identifying how many individuals can be accommodated and where they should be seated in order to maintain appropriate/required physical distancing.

$$Maximize\;\sum_{j}X_{j}$$ (1)

$$Subject\;to\;X_{j}+X_{j^{\prime }}\leq 1\;{\forall j,j}^{\prime }\in \Omega_{j}$$ (2)

$$X_{j}\in \{0,1\}\;\forall j$$ (3)

The model objective, (1), is to obtain the maximum number of individuals that can be seated in a classroom. This is the seating capacity. The primary constraints, (2), limit any two seats from being selected for individuals if they are too close to each other (see [8, 20] for alternative constraint forms). Constraints (3) stipulate that decision variables are binary, meaning that a seat is selected for usage or it is not. With a modification to (1) that includes the benefit/value of each node/vertex (a seat in this paper), Padberg [15] and Nemhauser and Trotter [16] refer to (1)-(3) as a node (or vertex) packing problem. Moon and Chaudhry [17] label (1)-(3) the anti-covering problem, though structure constraints (2) in a different form. In a spatial setting, the anti-covering location problem is most commonly encountered [8].

While there have been a number of efforts to explicitly formalize and model P-2, namely [18] and Erkut [19], the problem is generally conceived in terms of maximizing the distance between a seated individual and the individual closest to them. This then involves maximizing the minimum distance between individuals. It is possible to address P-2 by varying *R* using (1)-(3) such that the objective value, (1), is exactly equal to *p*, Specifically, one could search for *R*∈[*R*^*L*^, *R*^*U*^] where *R*^*L*^ results in (1) being less than *p* and *R*^*U*^ results in (1) being greater than *p*. The range [*R*^*L*^, *R*^*U*^] can then be systematically decreased until *R*∈[*R*^*L*^, *R*^*U*^] results in (1) being exactly equal to *p*. This is discussed in Church and Murray [8]. For the sake of simplicity, an additional model formulation is not detailed here since it is possible to accomplish the goals of P-2 through systematic variation of *R* along with evaluation, as will be demonstrated later in the paper.

A challenge with any spatial optimization model is application. As highlighted above, there are data input needs, but also an ability to solve the resulting application instance(s). In general terms, an optimization problem can theoretically be solved by exact or heuristic methods [23]. The human interactive approach described previously is an example of a heuristic, where a solution can be obtained using a devised, ad hoc approach, but there is no ability to prove whether the identified solution is optimal (the best possible) or even good with respect to the stipulated objective(s). The ability to formally prove solution quality is key, so any approach where the solution cannot be proven to be optimal is therefore a heuristic. In contrast, an exact approach can be proven to obtain the best (or optimal) solution under satisfied conditions. There exists commercial optimization software for solving integer programming problems along the lines of (1)-(3), though in practice successful solution is complicated by model structure, application size and computing capabilities that are a function of factors such as classroom size, number of potential seats and separation requirement.

## Classroom seating

**Fig 1:**
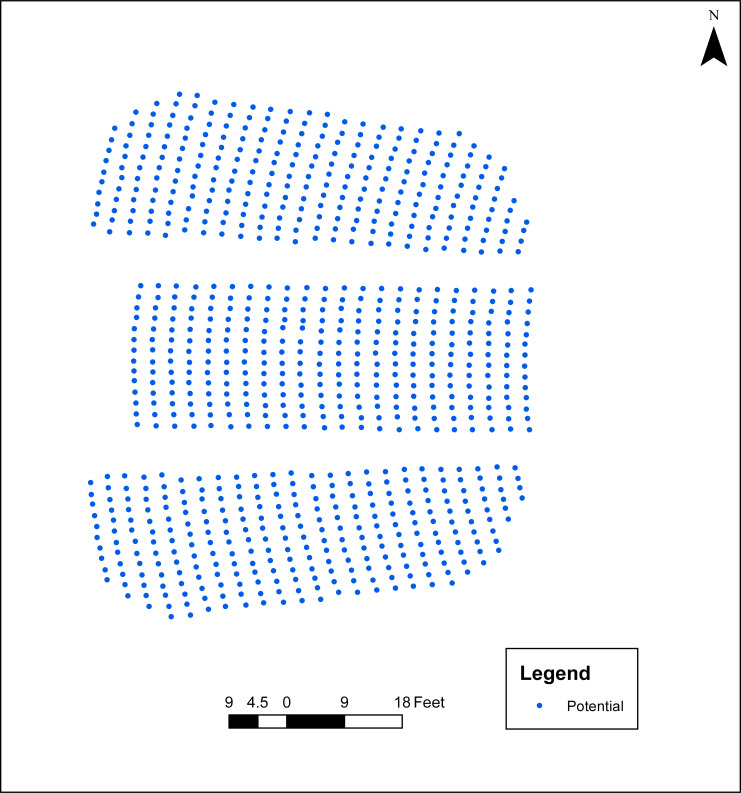
Potential seating in Campbell Hall.

Fixed position seating in a classroom is now explored. As a representative example, Campbell Hall at the UC Santa Barbara is considered [24]. Fig 1 shows the configuration and seating for Campbell Hall. It is the largest venue on campus with 867 seats, utilized for large course lectures, concerts and public events. The center of each seat was digitized (NAD 1983 State Plane California 5), with coordinates in feet. The restriction distance of 8.58333 ft. (8′ 7″) has been adopted by UC Santa Barbara as a physical distancing standard, taking into account the typical size of an individual (2.58333 ft., or 2′ 7″) along with the commonly accepted 6 ft. minimum (UC Santa Barbara 2020). Of course, safety interpretations may differ by organization. For example, Virginia Tech (2020) relies on a 6 ft. “nose-to-nose” separation between individuals. The ability to set a context specific standard is inherent in (1)-(3) through the use of an appropriate *R* for a given application instance. In order to illustrate modeling capabilities, all processing and computation is done on a desktop personal computer (Intel Xeon E5-2650 v3 CPU, 2.30 GHz with 96 GB RAM) running Windows 10 Pro (64-bit). Spatial optimization results are obtained using an exact solver, FICO Xpress (ver. 8.8), with all reported results confirmed to be optimal. Spatial data processing, evaluation, analysis and display / visualization were carried out using Esri ArcGIS (ver. 10.8).

Why is this a challenging problem to solve? One reason is simply due to the number of ways to seat individuals. In fact, the number of potential seating arrangements is 2^867^ = 9.84×10^260^, which is an extremely large number (in non-scientific notation, 984,025,245,785,089,668,542,779,581,465,789,315,325,854,953,445,720,527,559,445,628,946,039,644,175,627,938,424,082,949,548,321,194,750,581,672,402,577,714,396,421,513,631,013,567,628,805,598,203,955,105,649,136,727,087,197,834,782,616,666,551,076,670,067,483,604,388,140,535,333,015,300,386,199,264,902,135,056,251,471,428,268,545,287,733,116,928). Of course many arrangements are likely not feasible, violating the separation standard stipulated in Constraints (2), but technically all must be considered, explicitly or implicitly, in order to identify and conclude that a solution is optimal. Computationally, enumerating all seating arrangements is simply not possible, even with the most advanced supercomputing. Fortunately, structured approaches in integer programming can be relied upon for exact solution in this case.

**Fig 2:**
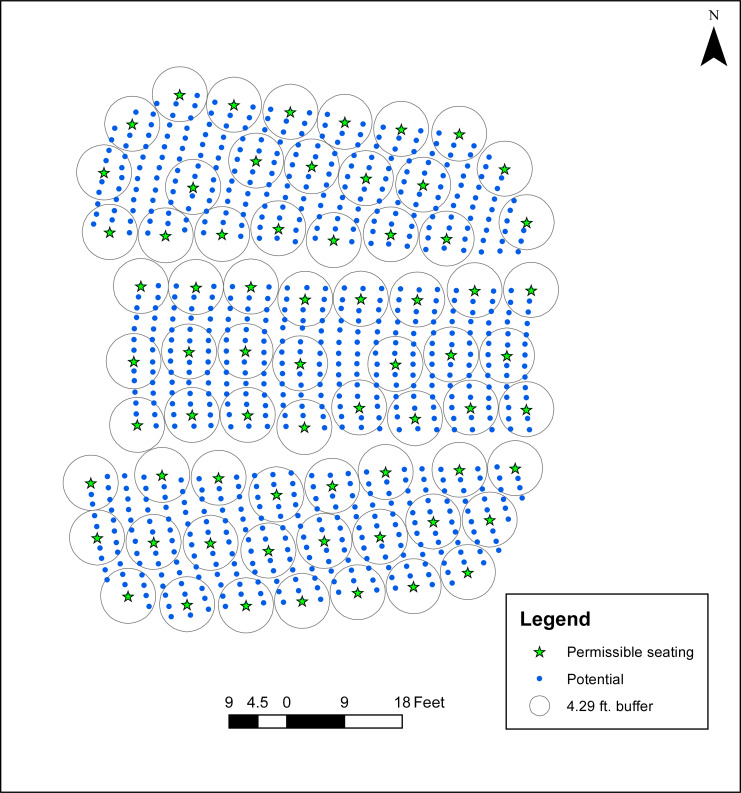
Maximum seating in Campbell Hall given physical distancing restriction (8.5833 ft).

Application of (1)-(3) to address the case of P-1 indicates that 68 individuals can be accommodated in Campbell Hall under the *R* = 8.5833 (8′ 7″) physical distancing standard adopted by University of California at Santa Barbara (see Table 1). This seating arrangement is shown in Fig 2, with a circle of radius 4.291667 ft. around each selected seat. This corresponds to R2, making it visually evident that the physical distancing standard is maintained as there are no intersecting circles. Further, it is not possible to add any more seats without violating physical distancing. A summary of seating capacity evaluation is given in Table 1, denoting the applied physical distancing standard (*R*), resulting maximum of objective (1) and computational effort to solve (“Solution time”). For comparison purposes, results utilizing a number of alternative physical distancing standards is also included, ranging from *R* = 11.0 down to *R* = 6.0. Table 1 therefore demonstrates the impact of physical distancing on seating capacity, where a more conservative standard results in less capacity (*R* = 11.0 accommodates 44 individuals) than would be possible under more relaxed conditions (*R* = 6.0 accommodates 117 individuals). Additionally, computational effort is rather minimal in general (often less than 10 seconds), but some physical distancing instances can be more difficult to solve (i.e., *R* = 7.0 requires 35 seconds and *R* = 6.0 requires over 162 seconds).

**Table 1:** Campbell Hall seating capacity under alternative physical distancing standards (867 potential seats).

*R*	Maximum seating capacity, (1)	Solution time (sec)
11.0	44	17.375
10.5	47	15.750
10.0	50	27.689
9.5	55	20.875
9.0	60	30.171
8.5833	68	6.859
8.5	69	5.875
8.4167	70	8.906
8.3333	72	8.484
8.0	73	9.155
7.5	82	6.641
7.0	87	35.032
6.5	100	8.796
6.0	117	162.484

**Fig 3:**
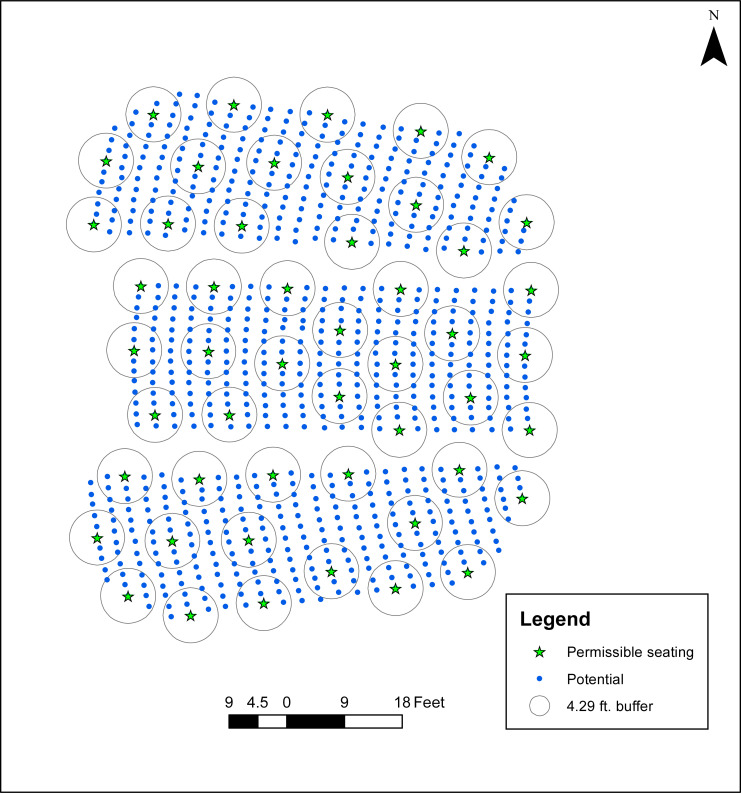
Dispersed seating of 50 individuals in Campbell Hall.

Turning the attention to the case of P-2, the University of California at Santa Barbara is also considering a maximum for any classroom of 50 individuals, irrespective of actual room size, in accordance with county guidelines [7]. To identify a seating arrangement where individuals are dispersed as much as possible from each other, totaling 50 (e.g., *p* = 50), the physical distancing standard was incrementally increased and the associated problem solved until *p* = 50 was found to be the maximum capacity (see Table 1). Shown in Fig 3 is the case where *R* = 10.0, with 50 individuals dispersed such that no two people are within 10 ft. of each other.

## Discussion

The seating analysis for Campbell Hall highlights how a formal spatial optimization model can be used to support assessment with respect to P-1 (capacity) and P-2 (spread apart) cases of dispersion. Solution time is relatively minimal on a personal computer, enabling flexibility to consider the impacts and implications of different separation standards. Further, this is supported by a generic model, applied by an analyst that need not be an expert in facilities management, layout design, etc. Of course, there are practical application issues that arise that are important to consider. First, some areas and seats may not actually be feasible. As an example, there are some 34 seats along the southeastern portion of Campbell Hall that are considered to have obstructed views when it is utilized for course lectures due to lectern and projection screen positioning (see Fig 2). Second, the point abstraction representing potential seat locations may not be positionally correct, either in absolute or relative terms. For Campbell Hall, assessment suggests that seat locations can only be considered accurate to within a few inches, but at this time it has not been possible to undertake any type of formal accuracy evaluation. Third, the ability to operationalize a seating plan that seeks to accommodate a maximum capacity requires precise implementation. That is, the maximum identified capacity will not be achieved by chance. Each of these items will be expanded upon in the discussion that follows.

As noted above, there are 34 seats in Campbell Hall with obstructed views during course lectures. As a result, these seats are not feasible for selection. Removing these seats and re-running the optimization model for the various scenarios indicates that fewer individuals can now be accommodated (Table 2). For *R* = 8.5833 (8′ 7″) physical distancing, 65 individuals are the most possible (P-1). This decreased capacity is similar for all instances when comparing Table 2 to the maximum capacities found in Table 1. The implications for P-2 are that seating the indicated level of individuals (*p* = 50) is achieved with slightly less physical distancing (*R* = 9.85) when examining the results summarized in Table 2. A major point is simply that what is deemed feasible is critical because the assumption is that all potential seats are feasible and equally viable in this case. While an important issue with outcome implication, this is readily accommodated in the framework detailed in this paper.

**Table 2:** Campbell Hall seating capacity accounting for view obstructions(833 potential seats).

*R*	Maximum seating capacity, (1)	Worst case capacity*	Individual selection capacity
			[min, median, max]
11.0	43	18	[24, 29, 35]
10.5	46	19	[25, 31, 37]
10.0	49	20	[28, 34, 40]
9.85	50	21	[28, 34, 41]
9.5	53	21	[29, 36, 42]
9.0	58	24	[34, 40, 47]
8.5833	65	29	[38, 46, 54]
8.5	67	27	[40, 47, 56]
8.4167	68	27	[39, 48, 56]
8.3333	70	28	[41, 49, 56]
8.0	71	29	[41, 51, 59]
7.5	80	32	[46, 54, 62]
7.0	84	37	[52, 60, 69]
6.5	97	41	[58, 68, 76]
6.0	113	50	[73, 81, 91]

* Obtained by solving the disruptive extension of (1)-(3) (see S1 Appendix)

**Fig 4:**
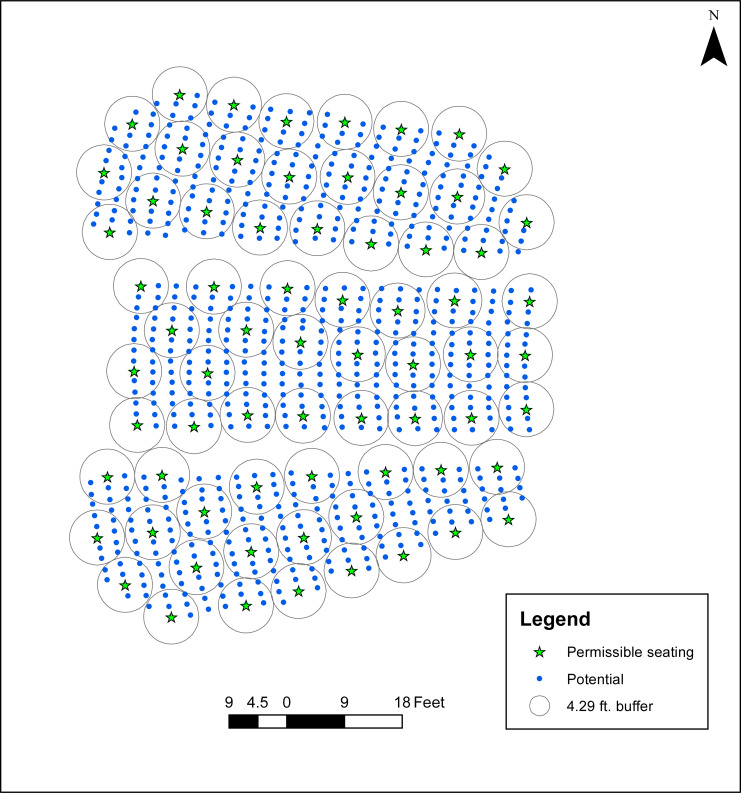
Maximum seating accounting for view obstructions (*R* = 8.3333 ft., with separation buffers of 8.5833 ÷ 2 = 4.29165 ft.).

The consideration of data quality, and in particular the accuracy of seat locations, is an important one. There has in fact been research exploring the significance of spatial data uncertainty in modeling efforts, such as the work of Murray [25]. Further, Wei and Murray [21] and Murray et al. [10] examined dispersion models where objects were represented as polygons, not points as is the case in this paper. Formal approaches have been developed to address various aspects of data uncertainty through model extensions. Given that potential seat locations are represented as points, one possible way to explore this issue here is by a systematic decrease of the physical distancing standard *R*. As an example, if the seat locations are considered to be within 3 inches of their actual location, then the use of *R* = 8.3333 ft. indirectly accounts for uncertainty. As indicated in Table 2, this instance has a seating capacity of 70 individuals. The seating arrangement is shown in Fig 4, with the circular area around each seat corresponding to 4.29165 ft., which is half of the more restrictive 8.5833 ft. standard. Fig 4 highlights that in this case there are but a few instances with at most minor overlaps. Such overlaps are likely within the confidence of the spatial data. This means that in practice they may in fact satisfy the physical distancing standard. Alternatively, a slight overlap may be deemed insignificant and permissible in practice.

**Fig 5:**
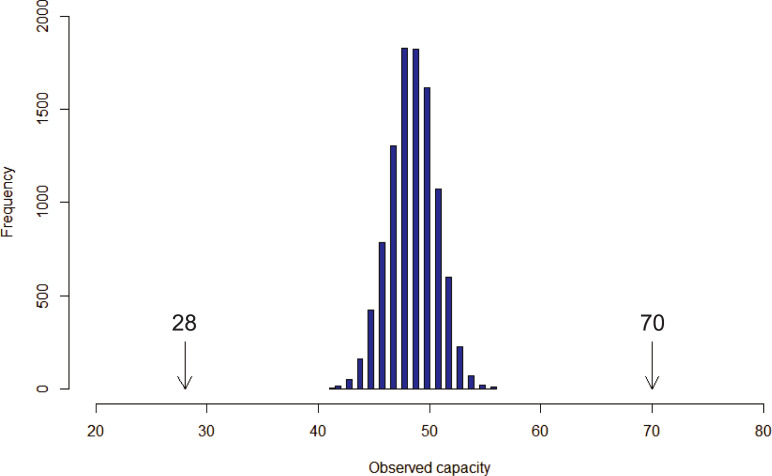
Distribution of resulting seating capacity assuming random individual selection (n = 10,000) for ***R***=8.3333 in Table 2.

The maximum identified capacity is the byproduct of applying a spatial optimization model, (1)-(3). It corresponds to a spatial arrangement of seating. Any deviation of this arrangement will invariably decrease capacity. Niblett and Church [22] introduced an extension of (1)-(3) to identify the worst case seating capacity where no additional individuals could be seated without violating the physical distancing standard (see S1 Appendix). This is particularly insightful information to the planning process. Table 2 includes this supplemental information under the “Worst case capacity” column. Additionally, information is provided regarding expected outcomes with respect to the capacity of individuals that select seats themselves in accordance with maintaining physical distancing. Specifically, 10,000 simulated seat selection scenarios are generated for each physical distancing standard (implemented in R). The simulation process selects an available seat at random for an individual, then makes all seats within the physical distancing standard unavailable for selection by others. The process repeats until all available seats have been selected. The total number of selected seats is then reported, and represents the realized capacity for that scenario. This assumes random arrival and seat selection by individuals, while maintaining necessary distancing. What is found is that expected classroom capacity would be substantially reduced. As an example, for *R* = 8.3333 the maximum capacity of 70 identified using (1)-(3) could be as low as 28 in the worst case, with the simulation of individual seating selection resulting in a capacity between 41 and 56, with a median of 49 as shown in the “Individual selection capacity” column of Table 2. This distribution is shown in Fig 5. For completeness, the capacity simulation results required 1.52 hours of computation time in this case, with the other identified instances in Table 2 ranging from 1.34 to 2.36 hours. It is clear from Table 2 and Fig 5 that achieving the maximum capacity is not something that will happen without precise planning detail as this is a challenging problem to solve. The obtained results are therefore significant because they are a byproduct of the process and framework as the optimal solution has great meaning.

## Conclusions

COVID-19 has required substantial re-thinking and re-evaluation of most activities. One of these is gatherings that bring together people, something that facilitates the spread of COVID-19. Given that a major challenge continues to be avoiding close contact between people, planning for social or physical distancing is critical. The continued return to and ramping up of in-person activities, whether it be work, education or entertainment, is dependent on structured situations that allows for people to be together in a safe way, enabled by physical distancing and mask wearing along with cleaning/disinfection measures. The paper highlights how planning for classroom physical distancing is possible, utilizing a spatial optimization model and supporting spatial analytics to develop a seating arrangement that minimize risk of COVID-19 spread. Further, two types of dispersion goals were addressed: deriving the maximum capacity of a classroom (P-1) and identifying an arrangement that provides for the most spacing possible between individuals in a classroom (P-2). Interestingly, both problem contexts can be addressed using (1)-(3) through strategic variation in parameter settings.

The Campbell Hall findings are insightful in many ways. The maximum capacity of 68 for *R* = 8.5833, the current physical distancing standard employed, in Table 1 may be only a starting point. It turns out that some seats are not feasible, with obstructed views. Further, the point abstraction representing potential seat locations appears to not be positionally correct, perhaps only accurate to within a few inches. Accordingly, the configuration showing a maximum capacity of 70 shown in Fig 4 (and summarized in Table 2, *R* = 8.3333 ft.) may be more representative of what is actually feasible in practice. Finally, operationalized seating plans associated with achieving a maximum capacity require precise implementation. The simulated individual seating and derived worst case capacity bounds summarized in Table 2 demonstrate that maximum identified capacity will not be achieved by chance.

The physical distancing accommodations addressed here reflect certain types of dispersion scenarios. The COVID-19 pandemic is highlighting that a range of other problem / planning variants are indeed important, yet remain research challenges for spatial analytics. Among the most interesting may be accounting for groupings of individuals from the same household or social bubble, effectively allowing for a relaxation of physical distancing in some situations.

## Supporting information

S1 Appendix (DOCX)
