# Supplementary material for: Planning for classroom physical distancing to minimize the threat of COVID-19 disease spread
Source: PLoS One. 2020 Dec 4;15(12):e0243345. doi: 10.1371/journal.pone.0243345 (PMC7717562; doi:10.1371/journal.pone.0243345)
Supplement: S1 Appendix — (DOCX) [file pone.0243345.s001.docx]

# S1 Appendix

As mentioned previously, the formulation (1)-(3) is often referred to as the anti-covering location problem due to Moon and Chaudhry [17], though other naming conventions also exist. The essence of (1)-(3) is finding the maximum capacity of an area to accommodate activity without violating physical distancing requirements. This may be viewed as an upper bound on what is possible. Unless its prescription is followed precisely, achieving such a bound is not likely, as demonstrated through the simulation results summarized in Table 2. Niblett and Church [22] extended (1)-(3) to identify a lower bound on capacity such that no additional activity could be accommodated. In this case, it would mean that no individuals could be seated without violating physical distancing. Niblett and Church [22] called this the disruptive anti-covering location problem. The formulation relies on the notation already introduced.

*Minimize* $\sum_{j} X_{j}$ (A-1)

*Subject to* $X_{j}+X_{j^{'}}\leq1 {\forall j,j}^{'}\in\Omega_{j}$ (A-2)

$X_{j}+\sum_{j^{'}\in\Omega_{j}} X_{j^{'}}\geq1 \forall j$ (A-3)

$X_{j}\in\left\{ 0,1 \right\} \forall j$ (A-4)

The objective, (A-1), is to identify the minimum number of individuals that can be seated in a classroom. Constraints (A-2) limit any two seats from being selected for individuals if they are too close to each other. Constraints (A-3) ensures that individuals are assigned to seats if at all possible. Constraints (A-4) impose binary conditions on decision variables.

Model (A-1)-(A-4) identifies the lower bound on capacity because the objective seeks the minimum. Of course, this alone is not enough because no assignment of individuals would be zero. However, no individuals are seated, so this is not of any significance from a capacity planning and management perspective. Thus, the combination of constraints (A-3) ensures that individuals are seated as long as physical distancing is possible, which is imposed constraints (A-2).
